# Redox-Responsive Comparison of Diselenide and Disulfide Core-Cross-Linked Micelles for Drug Delivery Application

**DOI:** 10.3390/pharmaceutics15041159

**Published:** 2023-04-06

**Authors:** Sonyabapu Yadav, Kalyan Ramesh, Obireddy Sreekanth Reddy, Viswanathan Karthika, Parveen Kumar, Sung-Han Jo, Seong II Yoo, Sang-Hyug Park, Kwon Taek Lim

**Affiliations:** 1 Department of Smart Green Technology Engineering, Pukyong National University, Busan 48513, Republic of Korea; 2 R&D Center, Devens Lab, SEQENS (CDMO) Pharmaceutical Solutions, Devens, MA 01434, USA; 3 Major of Display Semiconductor Engineering, Pukyong National University, Busan 48513, Republic of Korea; 4 Department of Biomedical Engineering, Pukyong National University, Busan 48513, Republic of Korea; 5 Department of Polymer Engineering, Pukyong National University, Busan 48513, Republic of Korea

**Keywords:** Diels–Alder reaction, disulfide, diselenide, core-cross-linked micelles, redox-responsive, drug delivery

## Abstract

In this study, diselenide (Se–Se) and disulfide (S–S) redox-responsive core-cross-linked (CCL) micelles were synthesized using poly(ethylene oxide)_2k_-*b*-poly(furfuryl methacrylate)_1.5k_ (PEO_2k_-*b*-PFMA_1.5k_), and their redox sensitivity was compared. A single electron transfer-living radical polymerization technique was used to prepare PEO_2k_-*b*-PFMA_1.5k_ from FMA monomers and PEO_2k_-Br initiators. An anti-cancer drug, doxorubicin (DOX), was incorporated into PFMA hydrophobic parts of the polymeric micelles, which were then cross-linked with maleimide cross-linkers, 1,6-bis(maleimide) hexane, dithiobis(maleimido) ethane and diselenobis(maleimido) ethane via Diels–Alder reaction. Under physiological conditions, the structural stability of both S–S and Se–Se CCL micelles was maintained; however, treatments with 10 mM GSH induced redox-responsive de-cross-linking of S–S and Se–Se bonds. In contrast, the S–S bond was intact in the presence of 100 mM H_2_O_2,_ while the Se–Se bond underwent de-crosslinking upon the treatment. DLS studies revealed that the size and PDI of (PEO_2k_-*b*-PFMA_1.5k_-Se)_2_ micelles varied more significantly in response to changes in the redox environment than (PEO_2k_-*b*-PFMA_1.5k_-S)_2_ micelles. In vitro release studies showed that the developed micelles had a lower drug release rate at pH 7.4, whereas a higher release was observed at pH 5.0 (tumor environment). The micelles were non-toxic against HEK-293 normal cells, which revealed that they could be safe for use. Nevertheless, DOX-loaded S–S/Se–Se CCL micelles exhibited potent cytotoxicity against BT-20 cancer cells. Based on these results, the (PEO_2k_-*b*-PFMA_1.5k-_Se)_2_ micelles can be more sensitive drug carriers than (PEO_2k_-*b*-PFMA_1.5k_-S)_2_ micelles.

## 1. Introduction

Chemotherapy is an effective method for treating cancer [1,2]. However, many anticancer drug molecules (paclitaxel, doxorubicin, and camptothecin) have unfavorable pharmacokinetic properties, such as potent toxicity, low water solubility, and a lack of selectivity [3,4]. To address these issues, scientists have developed nanoscale drug delivery carriers that include polymeric nano-carriers, liposomes, micelles, and dendrimers. Among all nano-carriers, polymeric micelles are gaining popularity because of their numerous benefits for drug delivery, such as improved aqueous solubility, prolonged drug retention time in plasma, low toxicity, and selective enhancement in a tumor area via the enhanced permeability and retention (EPR) effect [5]. Over the past several years, extensive research has been conducted on the use of polymeric micelles for the controlled administration of anticancer molecules and bio-imaging [6,7]. In addition, due to their unique properties, such as being able to respond to different stimuli at the same time and preferentially accumulating at the site of a tumor through the EPR effect, polymeric micelle carriers that can respond to different stimuli could be useful in a number of ways [8,9]. However, the concentration of anticancer compounds released into the cytoplasm by the nano-carriers is insufficient for their effectiveness. Therefore, it is important that anticancer drugs be rapidly released from micelles once they reach the cytoplasm to improve anticancer efficacy and reduce drug resistance in tumor cells [5,10]. Another disadvantage of conventional polymeric micelles is that they frequently experience early drug leakage due to their low physical stability in vivo and inadequate intracellular drug release at tumor locations, thereby restricting their clinical uses.

Core-cross-linked (CCL) micelles, on the other hand, have gained a lot of attention in the drug delivery field recently because of the many benefits they offer over conventional polymeric micelles, including improved colloidal stability, biocompatibility, drug-loading, stimuli control, and prolonged drug release [11,12,13]. The most common techniques for generating CCL micelles include radical polymerization, disulfide bridges, photo cross-linking, bifunctional cross-linkers, and the Diels–Alder (DA) reaction [14,15,16,17,18]. The DA reaction has several advantages over other methods since DA is yet another metal-free click reaction. In addition, the DA reaction can be easily carried out in the water, so it is not required to use expensive or potentially toxic solvents [19,20]. It is interesting to note that the reaction speeds up significantly when it is carried out in the water because of the increased hydrogen bonding to the activated complex as well as the increased hydrophobic contacts between the reactants [21,22,23]. Over the last few years, NIR-responsive micelles have been developed for biomedical applications. These NIR-responsive carriers require NIR-responsive moieties and external stimuli for the release of bioactive agents to the target sites [16,24,25]. However, internal stimuli-responsive polymeric carriers have gained popularity because of their ability to respond to low-intensity stimuli (including pH and redox) and deliver drugs on demand at tumor locations or within tumor cells. In particular, redox-responsive carriers are appealing due to their ability to enhance drug release at tumor locations and inside tumor cells in response to increased glutathione (GSH) concentrations relative to normal physiological conditions. Furthermore, these stimuli-responsive carriers are not dependent on external stimuli or NIR-responsive moieties [26,27,28,29].

GSH concentrations in tumor tissue are 100–1000 times higher than those in normal blood and extracellular fluid, and intracellular GSH concentrations in tumor cells are 2–10 mM, providing redox features to tumor cells [30]. This one-of-a-kind intracellular redox potential allows for the development of flexible, internal stimuli-responsive polymeric carriers with improved intracellular drug delivery, which in turn, boosts the therapeutic efficacy. GSH stimuli-responsive linkages, such as disulfide and diselenide linkages, are introduced in polymeric drug carriers to achieve this strategy [31]. Disulfide moieties may play a significant role in the conversion of molecular oxygen to reactive oxygen species (ROS), which are engaged in a number of essential biological reactions [32]. In the past few years, progress has been made in the use of disulfide linkage-using drug delivery systems for induced intracellular drug release. GSH, a reductive agent, can swiftly break the disulfide connection of the polymeric micelles by the thiol-disulfide conversion [33,34,35]. According to reports, various tumor cells have varying amounts of GSH, so the disulfide-containing carrier doesn’t show much effect on the tumor cells that have a low GSH [28,36]. Therefore, it is important to identify alternative redox-sensitive connections with greater sensitivity in polymeric micelles in order to stimulate effective drug release in tumor cells with low GSH.

In recent years, the diselenide bond has been considered the most appealing redox-responsive cross-links due to its various benefits, such as its hydrolytic stability under physiological conditions and its low-bond energy (172 kJ/mol) relative to that of the disulfide bond (268 kJ/mol) [37,38]. The diselenide bond has a lower energy, which makes it more amenable to oxidation in the presence of hydrogen peroxide (H_2_O_2_) during the phase conversion from hydrophobic diselenide to hydrophilic selenic acid [39]. H_2_O_2_ is often the most prevalent and persistent non-radical ROS found in cells. In normal tissue, the concentration of H_2_O_2_ is closely limited to 20 nM; however, in cancer tissue, it can reach 50–100 μM due to excessive H_2_O_2_ synthesis and storage [40]. Thus, both ROS and GSH are capable of cleaving the Se–Se bond [31]. Because of their stimuli responsiveness and high biocompatibility, Se-containing compounds have found widespread use in the development of stimuli-responsive carriers for biomedical applications.

**Scheme 1:**
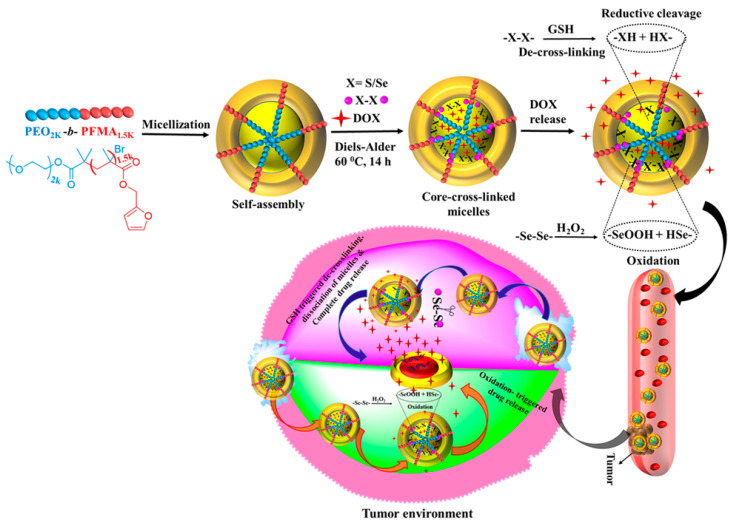
Schematic representation of DOX release under the conditions of GSH and H_2_O_2_ and the synthesis of CCL micelles using PEO_2k_-*b*-PFMA_1.5k_ block copolymers.

Herein, we have prepared (PEO_2k_-*b*-PFMA_1.5k_-C)_2,_ (PEO_2k_-*b*-PFMA_1.5k_-S)_2,_ and (PEO_2k_-*b*-PFMA_1.5k_-Se)_2_ (i.e., C–C/S–S/Se–Se) CCL micelles of PEO_2k_-*b*-PFMA_1.5k_ copolymers using maleimide-containing cross-linkers, such as 1,6-bis(maleimide) hexane (BisMH), dithiobis(maleimido)ethane (DTME), and diselenobis(maleimido)ethane (DseME), in order to compare their redox-responsive reactivity [16,41,42]. The hydrophilic PEO structure makes it biocompatible, biodegradable, and resistant to nonspecific absorption. On the other hand, the hydrophobic PFMA cores can hold drug molecules by the hydrophobic-hydrophobic interaction and also provide a pathway for the DA cycloaddition reaction with BisMH, DTME, and DseME cross-linkers. The size and PDI of C–C/S–S/Se–Se CCL micelles and non-CCL micelles were investigated before and after GSH/H_2_O_2_ treatment. In vitro release behavior of the micelles was compared in different pHs and redox environments [43]. In vitro cytotoxicity of non-CCL, C–C/S–S/Se–Se CCL, and DOX-loaded C–C/S–S/Se–Se CCL micelles was tested against HEK-293 and BT-20 cells. Furthermore, the cellular internalization of free DOX and DOX-loaded C–C/S–S/Se–Se CCL micelles into BT-20 cells was examined in order to evaluate their capabilities as redox-sensitive drug delivery carriers (Scheme 1).

## 2. Materials and Methods

### 2.1. Synthesis of BisMH, DTME, and DseME Cross-Linkers

**Figure 1:**
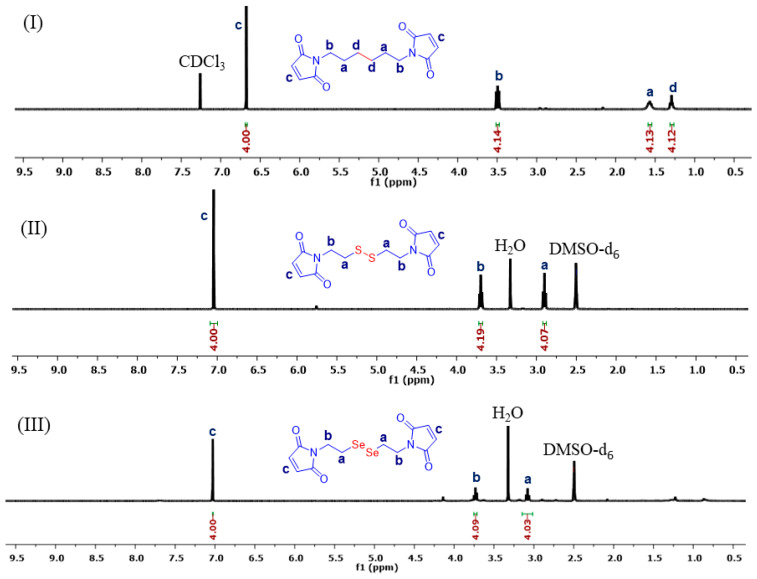
^1^H NMR of (**I**) BisMH in CDCl_3_, (**II**) DTME, and (**III**) DseME, cross-linker in DMSO-d_6_.

For the synthesis of DTME and DseME cross-linkers, the first step was to remove hydrochloric acid from cystamine dihydrochloride/selenocystamine dihydrochloride using the following procedure [41]. Cystamine dihydrochloride and selenocystamine dihydrochloride (1 g, 1 eq.) were suspended separately in methanol (10 mL). To this reaction mixture, KOH (0.547 g/0.386 g, 2.2 eq.) was added, and the white suspension was agitated for 20 h at ambient temperature. Afterward, the reaction mixture was filtered and concentrated to obtain residues. The obtained residues were diluted with dichloromethane (DCM) (20 mL) and washed with a saturated NaHCO_3_ solution (15 mL). The organic phase was dried over MgSO_4,_ and DCM was removed using a rotary evaporator to afford cystamine and selenocystamine. The 1,6-Hexanediamine, cystamine, and selenocystamine (0.500 g, 1 eq.) were dissolved separately in 5 mL of acetone. To this solution, 0.843 g, 0.643 g, and 0.398 g (2 eq.) of maleic anhydride were added, respectively. The immediate precipitation of the diacid was observed, and the reaction mixture was agitated for another hour in order to finish the reaction. To the reaction mixture, 0.598 mL, 0.456 mL, and 0.282 mL (1 eq.) of triethylamine were added, respectively. To this reaction mixture, sodium acetate (0.03 g) was added and heated slowly until it began to reflux, and at the same time, 1.098 mL, 0.838 mL, and 0.518 mL (2.7 eq.) of acetic anhydride were added correspondingly. The mixture was allowed to continue to reflux for an additional 3 h before the acetone was evaporated. Then, 15 mL of saturated NaHCO_3_ was added to the obtained residue, and the mixture was extracted with DCM (10 mL × 3) to get rid of the trimethylamine salt. The DCM phase was dried over MgSO_4_ and concentrated using a rotary evaporator. Azeotropic distillation with cyclohexane was utilized in order to eliminate the residual acetic anhydride in the reaction mixture. After that, a 2:3 mixture of ethyl acetate and hexane was used in column chromatography to remove the impurities from the crude. ^1^H-NMR (BisMH) (400 MHz, CDCl_3_, ppm): *δ* = 6.6 (s, 4H, 2-CH=CH-), 3.4 (t, 4H, N-CH_2_-CH_2_), 1.5 (t, 4H, CH_2_-CH_2_), and 1.2 (t, 4H, -CH_2_-CH_2_). ^1^H-NMR (DTME) (400 MHz, DMSO-d_6_, ppm): *δ* = 7.0 (s, 4H, 2-CH=CH-), 3.6 (t, 4H, N-CH_2_-CH_2_), and 2.8 (t, 4H, -CH_2_-CH_2_-S). ^1^H-NMR (DseME) (400 MHz, DMSO-d_6_, ppm): *δ* = 7.0 (s, 4H, 2-CH=CH-), 3.7(t, 4H, N-CH_2_-CH_2_), and 3.0 (t, 4H, Se-CH_2_-CH_2_) (Figure 1).

### 2.2. Synthesis of PEO_2k_-b-PFMA_1.5k_

FMA (0.645 mL, 9 eq.), PEO_2k_-Br [44] (1 g, 1 eq.), copper (Cu) wire (0.049 g, 8 eq.), tris[2-(dimethylamino) ethyl] amine (Me_6_TREN) (0.009 mL, 0.36 eq.), and dry dimethylformamide (DMF) (2 mL) were placed in a round bottom flask and stirred at room temperature in an N_2_ environment for 18 h. The molar ratio of reactants, [macro-initiator]:[monomers]:[Me_6_TREN]:[Cu-wire] was 1:9:0.36:8. The resulting product was purified using column chromatography with DCM as an eluent. The DCM solution was concentrated under reduced pressure, and the concentrate was transferred drop-by-drop into cold diethyl ether to get block copolymers as a white precipitate, which was then dried in a vacuum oven (0.950 g, 89.0%). ^1^H NMR (400 MHz, CDCl_3_, ppm): *δ* = 7.39 (s, 9H), 6.35–6.32 (d, 18H), 4.88–4.85 (d, 18H), 3.35 (s, 3H), 2.07 (s, 18H), 1.74 (s, 6H), 0.81 (s, 27H). M_n,GPC_ = 3900 g/mol.

### 2.3. Critical Micelle Concentration (CMC) Measurement

PEO_2k_-*b*-PFMA_1.5k_ concentrations ranging from 0.0001 to 1.0 mg/mL were prepared using deionized water. After dissolving pyrene in acetone, it was then added to each of the PEO_2k_-*b*-PFMA_1.5k_ concentrations in order to achieve a final pyrene concentration of 6 × 10^−7^ M in each sample. Afterward, the solution was left overnight to evaporate acetone. At an emission wavelength of 394 nm, the excitation spectra of samples (280–380 nm) were recorded with a slit width of 5 nm. The ratio of the peak intensity (*I*_337_/*I*_333_) of the excitation spectra was plotted as a function of the PEO_2k_-*b*-PFMA_1.5k_ concentration.

### 2.4. The Stability of Non-CCL/DOX and C–C/S–S/Se–Se CCL/DOX Micelles

The stability of non-CCL/DOX and C–C/S–S/Se–Se CCL/DOX micelles in PBS (pH 7.4) was examined using DLS. Micelles (1 mg/mL) were adjusted to buffer solutions, and their particle size was measured up to 12 d. In addition, we also performed the stability studies of non-CCL and C–C/S–S/Se–Se CCL micelles at different pH values of 7.2 and 6.5 and at different temperatures of 4 °C and 24 °C for 7 d (Figure S1). The Zeta potential of DOX-loaded micelles and free micelles was analyzed using a Zetasizer Nano-ZS instrument (Malvern, UK) (Figure S2).

### 2.5. Synthesis of DOX-Loaded C–C/S–S/Se–Se CCL Micelles of PEO_2k_-b-PFMA_1.5k_ via DA Reaction

PEO_2k_-*b*-PFMA_1.5k_ (10 mg, 1 eq.) and BisMH/DTME/DseME cross-linkers (2 mg/2.3 mg/3 mg, 3 eq.) were dissolved in 1 mL of acetonitrile, and an excess amount of water was added to the solution drop by drop while the mixture was being vigorously stirred. The resulting micellar solution was stirred for 14 h at a temperature of 60 °C in order to initiate cross-linking in the micellar core. The DLS technique was utilized to determine the size of the CCL micelles (1 mg/mL).

Before DOX-loading, hydrochloride was removed from DOX∙HCL by dissolving it with triethylamine in a 1:3 molar ratio into DMF and agitating the mixture at 22 °C for the whole night in a dark environment. In order to load DOX into the micellar core, PEO_2k_-*b*-PFMA_1.5k_ (10 mg, 1 eq.), BisMH/DTME/DseME cross-linker (2 mg/2.3 mg/3 mg, 3 eq.), and DOX (2.5 mg in 250 μL of DMF) were mixed in acetonitrile (1 mL); after that, 5 mL of PBS was added in a dropwise fashion while the mixture was vigorously stirred. The cross-linking was induced by stirring the mixture at 60 °C for 14 h, and the resulting C–C/S–S/Se–Se CCL/DOX micellar solution was subjected to dialysis (3.5 kDa) against deionized water, and fresh deionized water was replaced every 2 h for a day. Finally, the unloaded DOX was separated from the C–C/S–S/Se–Se CCL/DOX micelles by centrifugation at 2000 rpm for 5 min. A similar approach was used to prepare non-CCL/DOX micelles without the use of cross-linkers. The amount of DOX present in the micelles was determined by assaying the absorbance of DOX at λ_max_ of 485 nm using UV-Vis spectroscopy. The following formulas were used to determine the loading content (LC) and loading efficiency (LE).

$$\mathrm{LE}\;(\%)=\frac{\mathrm{Weight}\;\mathrm{of}\;\mathrm{loaded}\;\mathrm{DOX}}{\mathrm{Weight}\;\mathrm{of}\;\mathrm{DOX}\;\mathrm{used}}\;\times \;100$$

$$\mathrm{LC}\;(\%)=\frac{\mathrm{Weight}\;\mathrm{of}\;\mathrm{loaded}\;\mathrm{DOX}}{\mathrm{Total}\;\mathrm{weight}\;\mathrm{of}\;\mathrm{DOX}-\mathrm{loaded}\;\mathrm{micelles}}\;\times \;100$$

### 2.6. In Vitro Release Study of DOX from S–S/Se–Se CCL Micelles

In vitro DOX release study was carried out in response to redox stimuli (100 mM H_2_O_2_ and 10 mM GSH) in pH 7.4 and 5.0. 3 mL of DOX-loaded S–S/Se–Se CCL micelle solution (1 mg/mL) was dialyzed (3.5 kDa) against 15 mL acetate buffer solution (pH 5.0) and PBS (pH 7.4) with or without GSH/H_2_O_2_ at 37 °C at 100 rpm. The release behavior of non-CCL/DOX micelles in pH 7.4 and 5.0 was also investigated for comparison. At predetermined time intervals, 1 mL dialysate was withdrawn to measure DOX absorbance and replaced with fresh corresponding buffer solution (1 mL). The DOX released from S–S/Se–Se CCL/DOX and non-CCL/DOX micelles were determined using the cumulative method by measuring the DOX absorbance at 485 nm. The release data were fitted into the zero-order, first-order, Higuchi, and Korsmeyer–Peppas models to study the mechanism of DOX release from the micelles.

## 3. Results and Discussion

### 3.1. Synthesis and Characterization of C–C/S–S/Se–Se CCL Micelles

**Scheme 2:**
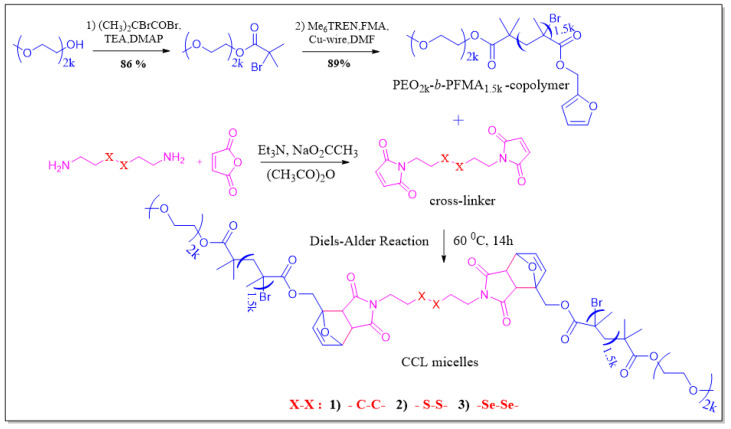
Preparation of cross-linkers, PEO_2k_-*b*-PFMA_1.5k_ copolymer, and C–C/S–S/Se–Se CCL micelles.

**Figure 2:**
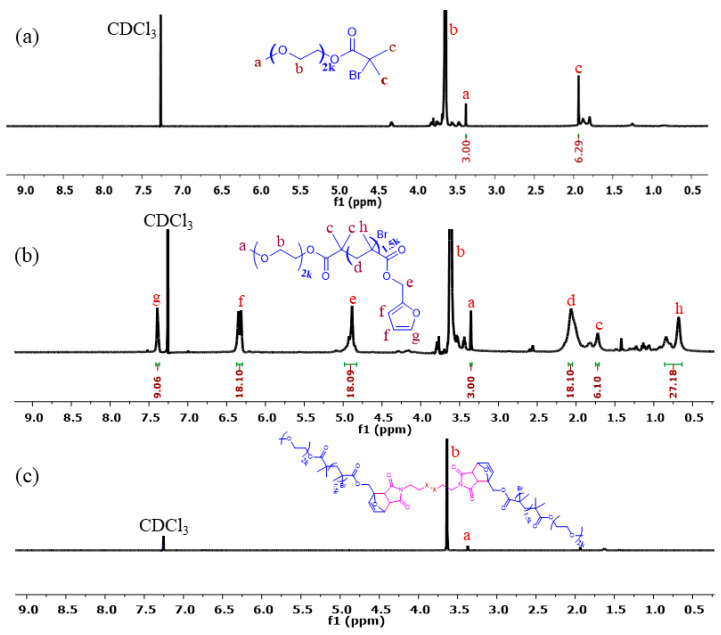
^1^H NMR of (**a**) PEO_2k_-Br, (**b**) PEO_2k_-*b*-PFMA_1.5k_, and (**c**) CCL micelles in CDCl_3_.

Cross-linkers play a crucial role in the development of stimuli-responsive carriers, and the disulfide/diselenide bond is a good candidate for a cross-linker because it has a low bond dissociation energy and is easy to oxidize in response to even weak stimuli. DTME/DseME cross-linkers were prepared by reacting cystamine/selenocystamine with maleic anhydride (Figure 1, Scheme 2), which were used to produce redox-responsive S–S/Se–Se CCL micelles via the DA reaction [20,41]. C–C/S–S/Se–Se CCL micelles of PEO_2k_-*b*-PFMA_1.5k_ were formed by dispersing PEO_2k_-*b*-PFMA_1.5k_ in water, followed by the addition of BisMH/DTME/DseME cross-linkers, respectively. The DA reaction was induced between the furfuryl units in the micellar core and the maleimide units of the cross-linker by applying heat. In the ^1^H NMR (CDCl_3_) spectra (Figure 2) of CCL micelles, the peaks associated with PEO_2k_ segments were present, but the peaks associated with FMA protons were not observed. This indicates that the cross-linked FMA segments in the cores of the micelles were unable to dissolve in the solvent, whereas the PEO segments that were present in the outer shell of the micelles were able to maintain their solvated structure.

**Figure 3:**
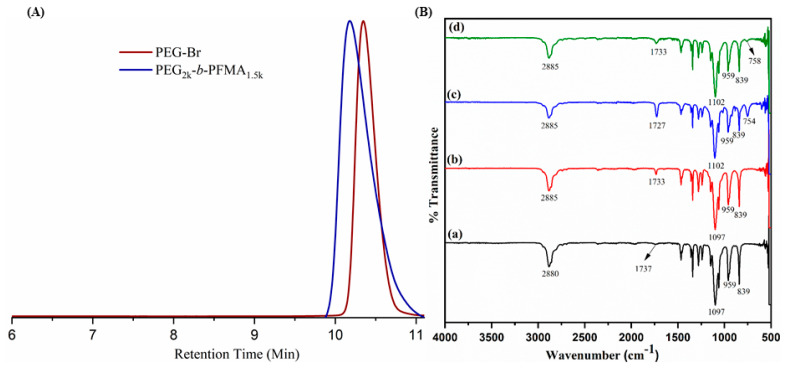
GPC chromatograms of the PEO_2k_-Br macro-initiator and PEO_2k_-*b*-PFMA_1.5k_ copolymer (**A**), FTIR spectra of (a) PEO_2k_, (b) PEO_2k_-Br, (c) PEO_2k_-*b*-PFMA_1.5k_, and (d) CCL (**B**).

The block copolymer of PEO_2k_-*b*-PFMA_1.5k_ was synthesized using single electron transfer-living radical polymerization (SET-LRP), which is the most popular method in polymerization for generating well-controlled linear segments with numerous benefits, including narrow molecular weight distribution, a homogeneous network, and intensive end-chain activity. In general, the size (strength) of hydrophilic (shell) and hydrophobic (core) parts determines the shape of micelles. In this study, we prepared an amphiphilic copolymer with a larger shell (hydrophilic) block size in order to generate micelles resembling spherical, star-like micelles. The hydrophilic part of the block polymer was constructed using the PEO_2k_-Br macro-initiator, and the size (~1500 g/mol) of the hydrophobic part was carefully controlled by the feeding ratio of FMA to the macro-initiator. Figure 3A depicts the symmetric and unimodal GPC curves of PEO_2k_-Br and PEO_2k_-*b*-PFMA_1.5k_. The evident shift of the GPC curve upon polymerization with a polydispersity (*Ð*) of 1.12 demonstrated that the polymerization process was well-controlled. FT-IR spectral analysis was used to analyze the block copolymer and CCL micelles, and the results are depicted in Figure 3B. The preparation of the PEO_2k_-Br macro-initiator from PEO is confirmed by the presence of vibration stretching bands of ester and C–O–C at 1097 and 1733 cm^−1^, respectively. The FTIR spectra of PEO_2k_-*b*-PFMA_1.5k_ shows similar bands to that of PEO_2k_-Br, along with a new band at 754 cm^−1^ attributed to the presence of C–H bending frequencies of furan rings, which confirms the generation of block polymer PEO_2k_-*b*-PFMA_1.5k_. The bands of PFMA at 1730 and 1111 cm^−1^ disappeared in the FTIR spectra of PEO_2k_-*b*-PFMA_1.5k_, which is due to the overlap of these bands with the stretching bands of the PEO_2k_-Br macro-initiator. The formation of CCL micelles using PEO_2k_-*b*-PFMA_1.5k_ via DA reaction is confirmed by the reduction of the C–H bending frequency at 754 cm^−1^ of the furan ring after interaction with the cross-linker.

^1^H NMR was used to examine the successful synthesis of PEO_2k_-Br, PEO_2k_-*b*-PFMA_1.5k,_ and CCL micelles of PEO_2k_-*b*-PFMA_1.5k_. The spectra of PEO_2k_-Br show the signals at 4.31–4.33, 3.37, and 1.93 ppm; these signals are assigned to protons of the methylene group (triplet) adjacent to the ester group, proton of terminal methoxy group of PEO (singlet), and protons of two methyl groups (singlet) (2-bromoisobutyryl moiety). Figure 2 shows the ^1^H NMR spectrum of the PEO_2k_-*b*-PFMA_1.5k_ copolymer, which reveals singlet methylene proton signals at 3.35 ppm from the PEO_2k_ moiety and triplet furfuryl proton signals at 7.39, 6.35–6.32, and 4.88–4.85 ppm from the PFMA moiety. From the GPC study, the *M*_n_ content was 3900 g mol^−1^, which was equivalent to the ^1^H NMR calculation of 3645 g mol^−1^ (Figure 2, Table 1).

### 3.2. Stability of C–C/S–S/Se–Se CCL/DOX and Non-CCL/DOX Micelles

**Figure 4:**
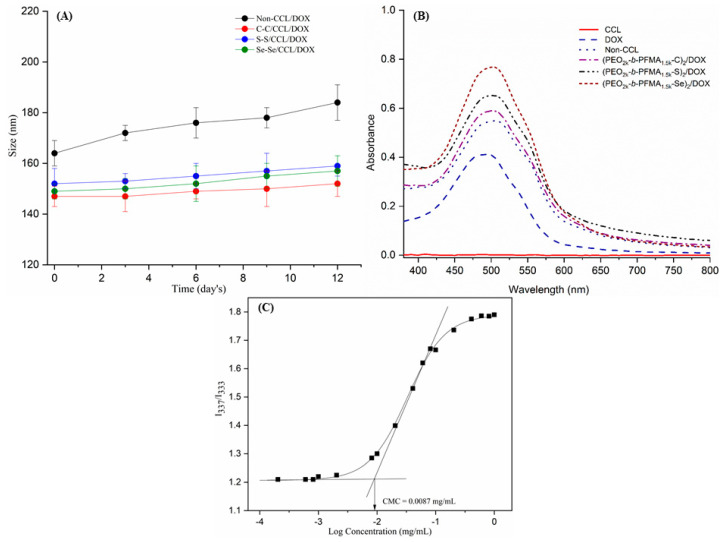
Stability of C–C/S–S/Se–Se CCL/DOX and non-CCL/DOX micelles (**A**), UV-Vis spectra of non-CCL/DOX, CCL, DOX, C–C/S–S/Se–Se CCL/DOX (**B**), and the plot of I_337_/I_333_ vs. the logarithm of PEO_2k_-*b*-PFMA_1.5k_ concentration (**C**).

The dispersion stability of polymeric nanocarriers in PBS is a key factor in making them more useful. The stability of C–C/S–S/Se–Se CCL/DOX and non-CCL/DOX micelles was examined by evaluating the changes in their particle size in PBS over a period of 12 d (Figure 4A). Since the particle size of C–C/S–S/Se–Se CCL/DOX micelles in PBS didn’t change over the course of 12 d, the colloidal stability was found to be good. There was some variation in particle size of C–C CCL/DOX, S–S CCL/DOX, and Se–Se CCL/DOX, which ranged from 152–159 nm, 149–155 nm, and 147–152 nm, respectively. The particle size of non-CCL/DOX fluctuated between 164–184 nm. As a result, the cross-linking in the CCL/DOX micelles is more likely to remain stable across a variety of media, which is helpful for their safe transport in the bloodstream of the body [45,46]. The size of non-CCL and C–C/S–S/Se–Se CCL micelles (Figure S1) at pH 7.2 and 6.5 and at 4 and 24 °C, did not show any variation for 7 d, indicating that the developed micelles were stable at some pH and temperature ranges. The zeta potential of all free CCL micelles is above −10 mV; therefore, negatively charged CCL micelles can have good protein resistance and long bloodstream circulation times. However, the zeta potential values of DOX-loaded CCL micelles decreased due to the electrostatic interaction of the amine groups in DOX and the carboxyl groups of the CCL micelles (Figure S2).

CMC is an essential measurement for determining the stability of the micelles and provides compelling evidence that block copolymers can self-assemble. The CMC of PEO_2k_-*b*-PFMA_1.5k_ was measured using pyrene, a fluorescence probe, with an excitation wavelength of 280–380 nm and an emission wavelength of 394 nm. Figure 4C shows a plot between the pyrene fluorescence excitation intensity ratio (*I*_337_/*I*_333_) and the concentration of PEO_2k_-*b*-PFMA_1.5k_. This plot showed that the CMC of PEO_2k_-*b*-PFMA_1.5k_ was 0.0087 mg/mL, indicating that micelles form at a very low concentration.

**Figure 5:**
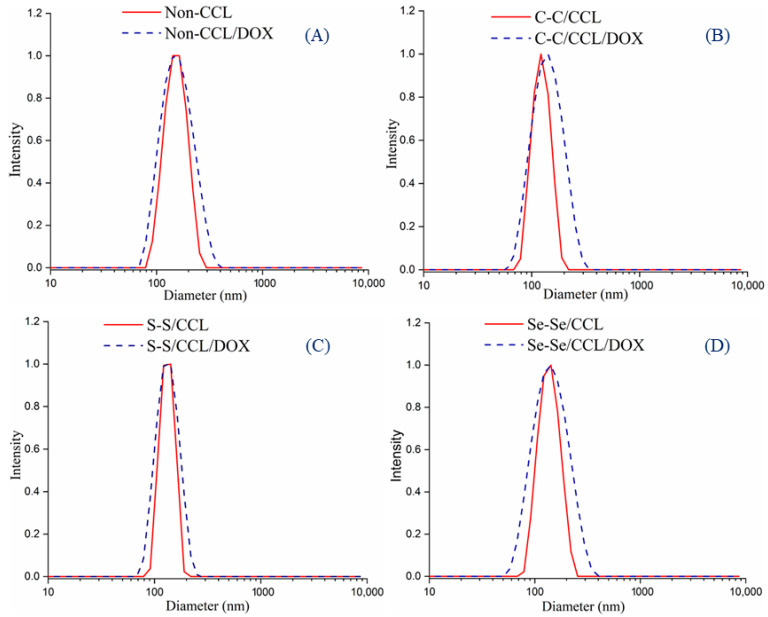
DLS profile of non-CCL, C–C/S–S/Se–Se CCL micelles with or without DOX (**A**–**D**).

The size and PDI of non-CCL, C–C/S–S/Se–Se CCL, C–C/S–S/Se–Se CCL/DOX, and non-CCL/DOX micelles were examined with DLS, and the results are shown in Figure 5. The sizes of C–C/S–S/Se–Se CCL micelles were determined to be 124 ± 35, 133 ± 31, and 139 ± 31 nm, respectively, and non-CCL micelles were measured to be 155 ± 35 nm, which are within the optimal range for the EPR effect [13,47,48]. After cross-linking, the micellar core formed a compact structure, which caused a decrease in the micellar size. The results are displayed in Table 2. The sizes of C–C/S–S/Se–Se CCL/DOX and non-CCL/DOX micelles were measured to be 147 ± 53, 152 ± 55, 149 ± 54, and 164 ± 54 nm, respectively, which indicated that the micellar size was marginally increased after DOX encapsulation.

### 3.3. Drug Loading Studies

DOX was used as a model drug to investigate the encapsulation capabilities of developed micelles, and the results are presented in Table 2. The unloaded DOX was removed by centrifuging the micellar solution at 2000 rpm for 5 min, followed by dialysis for 20 h. After loading, the characteristic peak of DOX (485 nm) was shifted to 505 nm, which is due to the generation of hydrogen bonding between DOX and the polymer core, indicating that DOX was successfully encapsulated into non-CCL and C–C/S–S/Se–Se CCL micelles. Figure 4B and Table 2 show that LC and LE of DOX into C–C/S–S/Se–Se CCL micelles are 7.76, 8.45, 9.03, and 68.36, 74.45, and 79.50%, respectively. On the other hand, the LC and LE of DOX in non-CCL micelles were determined to be 6.85 and 60.28%, respectively. The LC and LE of DOX in CCL micelles were higher than the non-CCL micelles, which is likely due to the stronger hydrophobic interactions between DOX and the cross-linked micellar core [49]. The core-cross-linking may also prevent the leakage of DOX from the CCL micelles while they are being dialyzed.

### 3.4. Redox-Responsive Properties of CCL Micelles

**Figure 6:**
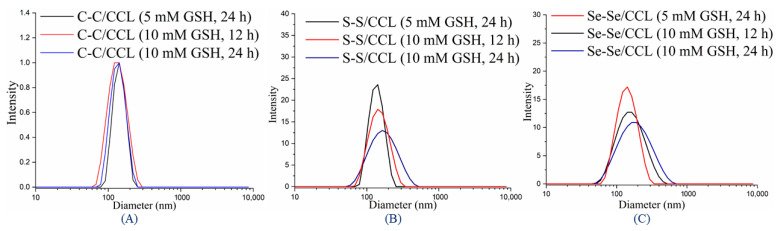
The size change of C–C/S–S/Se–Se CCL micelles with different GSH concentrations and treating time (**A**–**C**).

**Figure 7:**
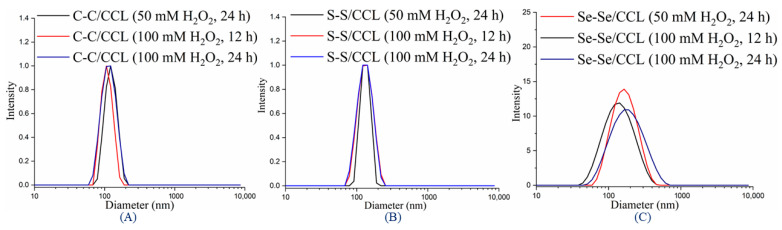
The size change of C–C/S–S/Se–Se CCL micelles with different H_2_O_2_ concentrations and treating times (**A**–**C**).

The redox-responsive behavior of C–C/S–S/Se–Se CCL micelles was investigated by observing their morphology and size changes under various redox environments. To simulate the conditions around tumor cells, we used GSH and H_2_O_2_ concentrations of 10 mM and 100 mM, respectively. During incubation with GSH and H_2_O_2_, the S–S/Se–Se CCL micelles underwent structural alterations, which were responsive to their reduced and oxidized states. As shown in Figure 6 (Table 3), S–S/Se–Se CCL micelles were swollen and widely dispersed after being treated with GSH, likely because of the cleavage of the disulfide and diselenide bonds at the cores, which resulted in the production of SH/SeH [50]. After being treated with GSH for 12 and 24 h at a concentration of 5 and 10 mM, the size of the S–S/Se–Se micelles increased. When S–S/Se–Se micelles were treated with H_2_O_2_ at a concentration of 50 and 100 mM for 12 and 24 h, the size of Se–Se CCL micelles also increased with broad dispersity, but the size of S–S CCL micelles did not change. This revealed that H_2_O_2_ was responsible for de-cross-linking Se–Se bonds at the core, resulting in the production of selenic acid (SeOOH) (Figure 7, Table 3) [51,52], but it did not affect the S–S bond. Whereas, in the case of C–C CCL micelles, there is no effect on the C–C bond when exposed to both GSH and H_2_O_2_ environments for 24 h, even when the concentration is increased to 100 mM because there is no change in the size of C–C micelles. From the above findings, it was concluded that the C–C bond does not cleave in a GSH or H_2_O_2_ environment, and the S–S bond can be cleaved only by GSH but not H_2_O_2._ On the other hand, both H_2_O_2_ and GSH cleave the Se–Se bonds present at the core of the micelles, allowing them to de-cross-link in response to the oxidation and reduction environment of tumors [53,54]. This result suggests that the diselenide-CCL micelles could be used as a more efficient candidate for tumor therapy.

### 3.5. Redox-Triggered In Vitro DOX Release

**Figure 8:**
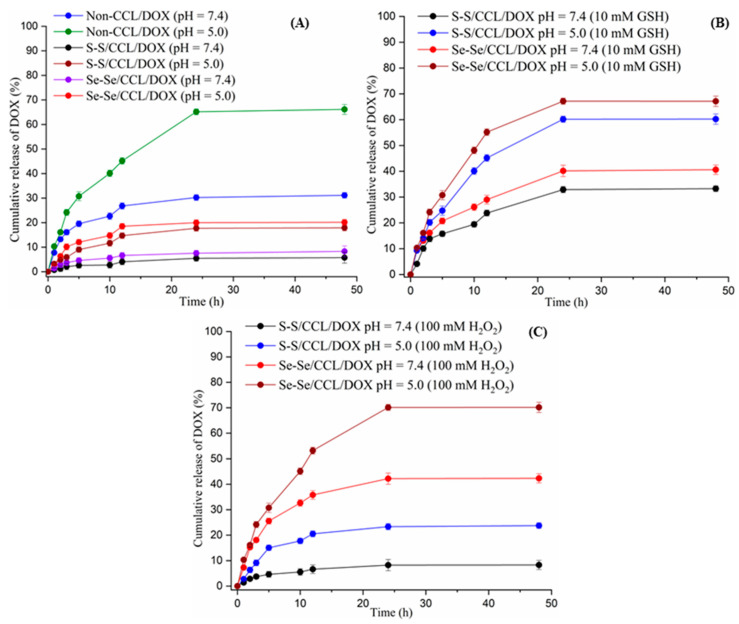
Cumulative release of DOX from non-CCL and CCL micelles at pH 5.0 and pH 7.4 (**A**), with GSH (**B**), and H_2_O_2_ (**C**).

The release behavior of DOX-loaded S–S/Se–Se CCL and non-CCL micelles were examined at two different pH values of 7.4 and 5.0 in different physiological environments (10 mM GSH and 100 mM H_2_O_2_), and the results are depicted in Figure 8. At pH 7.4 and 5.0, the release behavior of DOX-loaded non-CCL micelles showed 31 and 66% of DOX, respectively, whereas the DOX-loaded S–S/Se–Se CCL micelles showed lower release rates at both pH environments. When DOX-loaded S–S/Se–Se CCL micelles were exposed at pH 5.0 and 7.4 in the presence of a 10 mM GSH environment, the release rate of DOX-loaded S–S/Se–Se CCL micelles increased, and the cumulative release was more than 60% at pH 5.0 after 48 h, but the cumulative release was ~40% at pH 7.4 after 48 h. This is due to the fact that cleavage of the disulfide/diselenide bond of the CCL micelles takes place in the reduction environment of GSH. This result also indicates that the diselenide bond is more sensitive than the disulfide bond in a redox environment. The diselenide bond energy is low compared to that of the disulfide bond energy; hence, the diselenide bond cleaves easily in both environments and releases more DOX molecules. When DOX-loaded S–S/Se–Se CCL micelles were exposed to pH 5.0 and 7.4 in the presence of a 100 mM H_2_O_2_ environment, the cumulative release rate of DOX from Se–Se CCL micelles were 70% at pH 5.0 but a lower released amount at pH 7.4. On the other hand, the release rate of S–S CCL micelles was much lower than that of Se–Se CCL micelles in both pH environments (5.0 and 7.4). The release behavior was similar to those without a redox stimulus, indicating that the disulfide bond does not cleave in an H_2_O_2_ environment [28]. Compared to non-CCL/DOX micelles, CCL/DOX micelles release DOX slowly because the cross-linking protects the micellar core from DOX release. As a result of their minimal DOX leakage under normal physiological conditions, CCL micelles have an obvious advantage over non-CCL micelles. On the other hand, the DOX-loaded CCL micelles were expected to show significantly increased DOX release in tumor environments.

After fitting the release patterns of DOX-loaded micelles to different kinetic models, such as the zero-order, first-order, Higuchi, and Korsmeyer–Peppas models (see Table 4 for estimated regression coefficient (r^2^) values), the Higuchi model was found to be the best fit. This means that the buffer medium has to get into the micelle matrix in order for DOX to be released. In addition, the release exponents (n) were found to be in the range of 0.403 to 0.650 when computed with the Korsmeyer–Peppas equation. This indicates that the release of DOX from micelles is mostly mediated by a non-Fickian diffusion pattern, which means the polymer relaxation time is roughly equal to the typical solvent diffusion time.

### 3.6. Cytotoxicity of DOX-Loaded C–C/S–S/Se–Se CCL Micelles and Non-CCL Micelles

**Figure 9:**
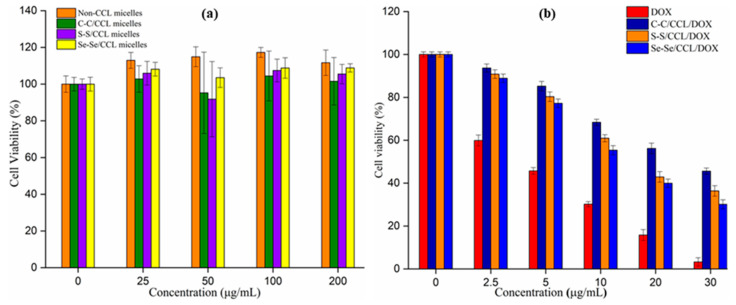
(**a**) Viability of normal HEK-293 cells after being exposed to blank non-CCL and C–C/S–S/Se–Se CCL micelles for 24 h and (**b**) viability of BT-20 cells following exposure to various concentrations of C–C/S–S/Se–Se CCL/DOX and free DOX micelles (24 h).

HEK-293 non-cancerous cells were used to investigate the biocompatibility of C–C/S–S/Se–Se CCL and non-CCL micelles. Blank non-CCL micelles and C–C/S–S/Se–Se CCL micelles demonstrated more than 85% HEK-293 cell survival in the presence of up to 200 µg/mL of micellar dosage (Figure 9a), indicating their exceptional compatibility with normal cells and high potential as nontoxic therapeutic carriers. In order to assess the C–C/S–S/Se–Se CCL/DOX micelles as a potential cancer therapy, their cytotoxic activity was also examined. The vitality of BT-20 cells was decreased in a concentration-dependent manner by C–C/S–S/Se–Se CCL/DOX micelles and free DOX (0. 2.5, 5, 10, 20, and 30 µg/mL of DOX concentration), suggesting a reduction in the growth of cancer cells (Figure 9b). Incomplete release of the encapsulated DOX from C–C/S–S/Se–Se CCL micelles is probably the reason for the reduced cell death of C–C/S–S/Se–Se CCL/DOX micelles (IC_50_: 24.98, 19.90, and 17.90 µg/mL) compared to free DOX (IC_50_: 8.39 µg/mL), which was comparable with drug-releasing results. In comparison to S–S DOX micelles, Se–Se DOX micelles had a lower half maximum inhibitory concentration (IC_50_) against BT-20 cells. When compared to disulfide micelles, diselenide micelles released more intracellular drugs. Se–Se CCL/DOX micelles caused 30% of BT-20 cell death at 30 µg/mL after 24 h, whereas C–C/S–S CCL/DOX micelles caused 36% at 30 µg/mL. Moreover, our findings imply that CCL/DOX micelles can cause BT-20 cell apoptosis.

**Figure 10:**
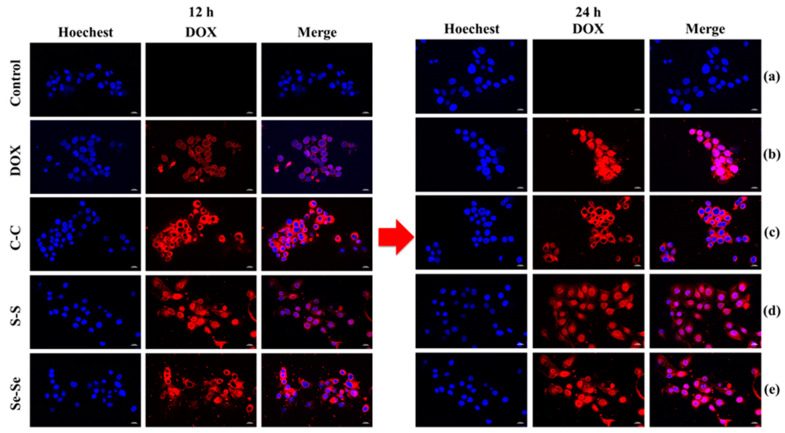
The CLSM images from left to right (each panel) display DOX fluorescence (red), BT-20 cell nuclei stained with DAPI (blue), and overlays of both images (merged). Non-treated cells (**a**), free DOX (**b**), and BT-20 cells treated with C–C/S–S/Se–Se CCL/DOX micelles (**c**–**e**) for 12 h and 24 h; the scale bar is 20 μm.

Using CLSM pictures, the cellular uptake of CCL micelles and intracellular DOX release patterns were studied. BT-20 cells were treated for 12 and 24 h with free DOX and C–C/S–S/Se–Se CCL/DOX micelles (DOX concentration: 0, 2.5, 5, 10, 20, and 30 µg/mL), respectively. As hypothesized, the untreated BT-20 cells had blue fluorescence in the nucleus but did not exhibit red fluorescence because DOX was not present (Figure 10a). Variable intracellular DOX distribution was seen at 12 and 24 h of incubation in C–C, S–S, and Se–Se CCL/DOX micelles and free DOX. After 12 h of incubation with free DOX, BT-20 cells showed bright red fluorescence at the nuclei (Figure 10b), and increased red intensity was seen after 24 h (Figure 10c), which revealed that most free DOX was quickly taken up by the cell organelles through the cell membrane and entered the cell nuclei. C–C CCL/DOX micelles showed a lesser red fluorescence that mostly stayed in the cytoplasm in contrast to free DOX. The endocytosis through which the C–C CCL/DOX micelles were absorbed by BT-20 cells is most probable to be responsible. Se–Se CCL/DOX micelles exhibit DOX fluorescence both in the endo/lysosome zone and in the nucleus, where it is substantially more intense than DOX-loaded disulfide micelles during the 12 and 24 h incubation times (Figure 10a,b,d). This is clearly triggered by the fact that more DOX penetrates the nucleus after being released from the DOX-loaded diselenide CCL micelles as a result of de-cross-linking in the redox environment [8,50,51]. The efficiency of Se–Se micelles is substantially greater than that of C–C and S–S micelles, as well as being similar to free DOX in tumor cell cellular uptake results. Overall, the findings showed that PEO_2k_-*b*-PFMA_1.5k-_based CCL/DOX micelles were capable of internalizing cancer cells and releasing the encapsulated DOX in the intracellular signaling pathways to trigger apoptosis.

## 4. Conclusions

Stimuli-responsive PEO_2k_-*b*-PFMA_1.5k_ CCL micelles were prepared using disulfide and diselenide-based cross-linkers, and their redox-responsive properties were examined. SET-LRP polymerization was used to prepare PEO_2k_-*b*-PFMA_1.5k_ copolymers with controlled molecular weight. The micellar cores of the copolymers were cross-linked with three different cross-linkers, such as BisMH/DTME/DseME, by the DA reaction to produce respective CCL micelles. The micelles formed with a narrow size distribution, and their sizes were less than 170 nm before and after core-cross-linking. The diselenide micelles showed significantly higher LC and LE values for DOX encapsulation. The LE values of C–C/S–S/Se–Se CCL micelles are 68.36%, 74.45%, and 79.50% of DOX, respectively. The in vitro release rate of DOX-loaded S–S/Se–Se CCL micelles showed a lower release rate at pH 7.4 and 5.0, but when micelles were treated with GSH (10 mM) and H_2_O_2_ (100 mM) at pH 5.0, the release rate of DOX-loaded S–S/Se–Se micelles was enhanced in the GSH environment. However, in the H_2_O_2_ environment, only the DOX-loaded Se–Se CCL micelles showed enhanced release rates but not the S–S CCL micelles. These results demonstrated that diselenide bonds are more susceptible than disulfide bonds. Both non-CCL and S–S/Se–Se CCL micelles demonstrated more than 85% cell viability on HEK-293 normal cells in in vitro cytotoxicity studies, indicating the biocompatibility of the micelles. In contrast, the drug-loaded micelles dramatically reduced the viability of cancer cells. The IC_50_ of Se–Se CCL/DOX micelles against BT-20 cells was lower than that of S–S CCL/DOX micelles. In BT-20 cells, there was a significant variance in the intracellular drug release from Se–Se CCL/DOX and S–S CCL/DOX micelles. From the confocal pictures, the CCL/DOX micelles showed endocytotic internalization into the cells, and disulfide micelles emitted less DOX than diselenide micelles did. Diselenide-containing CCL micelles offer a great deal of potential as intelligent carriers for anticancer drug delivery vehicles that can adapt to the tumor microenvironment.

## Figures and Tables

**Table 1:** Characteristic data of PEO_2k_-*b*-PFMA_1.5k_ copolymer.

Run	Polymer	Mn (NMR) ^a^ (g/mol)	Mn (GPC) ^b^ (g/mol)	*Ð*
1	PEO_2k_-Br	2150	2300	1.06
2	PEO_2k_-*b*-PFMA_1.5k_	3645	3900	1.12

^a^ Determined by ^1^H NMR., ^b^ Determined by GPC. Calibrated against PS standards (THF, 1 mL/min).

**Table 2:** Size, PDI, LC, and LE of prepared micelles before and after DOX encapsulation.

Micelles	Blank Micelles		DOX-Loaded Micelles ^b^			
	Diameter ^a^ (nm)	PDI ^a^	Diameter ^a^ (nm)	PDI ^a^	LC (%)	LE (%)
Non-CCL	155 ± 35	0.243	164 ± 54	0.261	60.28	6.85
C-C/CCL	124 ± 35	0.243	147 ± 53	0.237	68.36	7.76
S-S/CCL	133 ± 31	0.263	152 ± 55	0.260	74.45	8.45
Se-Se/CCL	139 ± 31	0.295	149 ± 54	0.251	79.50	9.03

^a^ Determined by DLS, ^b^ CCL/DOX micelles.

**Table 3:** Redox-responsive behavior of C–C/S–S/Se–Se CCL micelles with GSH and H_2_O_2_.

Micelles	Time (h)	5 mM GSH ^a^		50 mM H_2_O_2_ ^a^	
		Diameter (nm)	PDI	Diameter (nm)	PDI
C-C/CCL	24	124 ± 54	0.253	127 ± 54	0.277
S-S/CCL	24	141 ± 70	0.240	135 ± 31	0.293
Se-Se/CCL	24	156 ± 65	0.255	161 ± 54	0.327
**Micelles**		**10 mM GSH ^a^**		**100 mM H_2_O_2_ ^a^**	
		**Diameter (nm)**	**PDI**	**Diameter (nm)**	**PDI**
C-C/CCL	0	124 ± 35	0.243	--	--
	12	121 ± 57	0.248	125 ± 53	0.236
	24	126 ± 52	0.232	130 ± 52	0.211
S-S/CCL	0	133 ± 31	0.263	--	--
	12	148 ± 56	0.271	134 ± 64	0.271
	24	150 ± 75	0.355	137 ± 62	0.275
Se-Se/CCL	0	139 ± 31	0.295	--	--
	12	155 ± 61	0.293	159 ± 55	0.298
	24	158 ± 88	0.388	164 ± 69	0.322

^a^ CCL micelles.

**Table 4:** Release kinetic parameters of all formulations at pH 7.4 and 5.0.

Code	GSH/H_2_O_2_	pH	Zero-Order		First Order		Higuchi Model		Korsmeyer-Peppas	
			K_0_	r^2^	K_1_	r^2^	K_H_	r^2^	n	r^2^
Non-CCL/DOX	NA	7.4	0.932	1.558	0.013	1.036	5.966	0.475	0.403	0.959
		5.0	1.881	0.032	0.046	0.719	11.470	0.886	0.525	0.981
S-S/DOX	NA	7.4	0.160	0.206	0.002	0.239	0.964	0.895	0.548	0.884
		5.0	0.522	0.163	0.006	0.012	3.227	0.822	0.595	0.980
Se-Se/DOX	NA	7.4	0.239	0.648	0.003	0.568	1.497	0.734	0.476	0.955
		5.0	0.608	0.692	0.007	0.473	3.851	0.645	0.550	0.938
S-S/DOX	10 mM GSH	7.4	0.952	0.090	0.013	0.202	5.839	0.853	0.493	0.933
		5.0	1.739	0.063	0.039	0.672	10.618	0.871	0.631	0.997
Se-Se/DOX	10 mM GSH	7.4	1.173	0.426	0.018	0.049	7.270	0.809	0.432	0.994
		5.0	1.975	0.107	0.056	0.730	12.201	0.820	0.650	0.996
S-S/DOX	100 mM H_2_O_2_	7.4	0.255	1.031	0.003	0.933	1.622	0.590	0.515	0.994
		5.0	0.709	0.383	0.009	0.155	4.451	0.723	0.608	0.947
Se-Se/DOX	100 mM H_2_O_2_	7.4	1.466	0.326	0.028	0.284	9.152	0.770	0.605	0.992
		5.0	2.026	0.049	0.056	0.805	12.380	0.869	0.620	0.993

## Data Availability

The data presented in this study are available upon request from the corresponding author.

## Supplementary Materials

The following supporting information can be downloaded at: https://www.mdpi.com/article/10.3390/pharmaceutics15041159/s1. Martials and characterization techniques are in the supplementary information. Figure S1. Stability of C-C/S-S/Se-Se CCL and non-CCL micelles at pH = 6.5 (A) and pH = 7.2 (B) and 4 °C (C), 24 °C (D). Figure S2. Zeta potential measurement of Non-CCL and C-C/S-S/Se-Se CCL micelles with and without DOX.
