# Supplementary material for: Redox-Responsive Comparison of Diselenide and Disulfide Core-Cross-Linked Micelles for Drug Delivery Application
Source: Pharmaceutics. 2023 Apr 6;15(4):1159. doi: 10.3390/pharmaceutics15041159 (PMC10144204; doi:10.3390/pharmaceutics15041159)
Supplement: Supplementary file 1 [file pharmaceutics-15-01159-s001.zip › pharmaceutics-2282167-supplementary.pdf]

Supporting Information

Redox-Responsive Comparison of Diselenide and Disulfide Core-Cross-Linked Micelles for Drug Delivery Application

Sonyabapu Yadav [1], Kalyan Ramesh [2], Obireddy Sreekanth Reddy [3], Viswanathan Karthika [3], Parveen Kumar [3], Sung-Han Jo [4], Seong II Yoo [5], Sang-Hyug Park [4] and Kwon Taek Lim [1,3,]*

1 Department of Smart Green Technology Engineering, Pukyong National University, Busan 48513, Republic of Korea

2 R&D Center, Devens Lab, SEQENS (CDMO) Pharmaceutical Solutions, Devens, MA 01434, USA

3 Major of Display Semiconductor Engineering, Pukyong National University, Busan 48513, Republic of Korea

4 Department of Biomedical Engineering, Pukyong National University, Busan 48513, Republic of Korea

5 Department of Polymer Engineering, Pukyong National University, Busan 48513, Republic of Korea

* Correspondence: ktlim@pknu.ac.kr

Materials

Ethanol (EtOH), cystamine dihydrochloride, Selenocystamine dihydrochloride, 1,6-Hexanediamine, sodium borohydride ($NaBH_4$), poly (ethylene oxide) methyl ether (M_n = 2000 g mol^{-1}), selenium powder, 4-(dimethyl amino)pyridine (DMAP), glutathione (GSH), fetal bovine serum (FBS), furfuryl methacrylate (FMA), and maleic anhydride were purchased from Sigma-Aldrich (Seoul, South Korea). Anhydrous magnesium sulfate ($MgSO_4$), ammonium chloride (NH_4Cl), sodium acetate (NaOAc), triethyl amine (Et_3N), and potassium hydroxide (KOH) were purchased from Junsei Chemical Co., Ltd. Acetic anhydride (Ac_2O) was purchased from Fluka Chemicals. DOX hydrochloride (DOX·HCl) and 2-bromoisobutyryl bromide were obtained from Boryung Pharmaceutical Co. (Seoul, South Korea). Copper wire (0.5 mm) was purchased from Alfa-Aeser (Seoul, South Korea). WST assay kit was received from Daeil-Lab Services (Seoul,

South Korea). 4′,6-diamidino-2-phenylindole (DAPI) was purchased from Thermo Scientific (MA, USA). All the other mentioned solvents were obtained from Duksan Chemicals (Busan, South Korea).

Characterizations

NMR spectra were recorded on NMR spectrometer (JNM-ECP, JEOL, 400 MHz, Akishima, Japan). The molecular weight of block copolymers was determined by an Agilent 1200 Series GPC-SEC system (Agilent Technologies, USA) equipped with a RI detector and a PL gel column (102-104 Å; 5 μm) using THF as an eluent (calibrated against polystyrene standards) at 25 °C. The UV absorption spectra were measured on a UV/Vis-spectrophotometer (Optizen-POP, Daejeon, South Korea). The size distribution and hydrodynamic size of the micelles were determined by dynamic light scattering (DLS) using a Zetasizer Nano-ZS instrument (Malvern, UK) equipped with He-Ne laser (633 nm) and ELS controller at 90° for controlling optical measurements.

Synthesis of PEO_{2k}-Br macro-initiator

The PEO_{2k}-Br macro-initiator was synthesized accordance with the procedure described earlier [1]. DMAP (0.229g, 1.5 eq.) and TEA (0.174 mL, 1 eq.) were dissolved in 5 mL of DCM. To the stirred solution, 2-bromoisobutyryl bromide (0.174 mL, 1 eq.) in dry DCM was added at 0 °C. Thereafter, PEO_{2k} (4g, 1 eq.) in DCM (25 mL) was added drop-by-drop for 1 h under an N_2 environment to generate a yellow solution, after which the temperature was raised to 25 °C and the solution was stirred for 20 h. The reaction mixture was subjected to reduced pressure and concentrated until it was half its original volume. The solution was then precipitated in cold diethyl ether and re-precipitated twice with ethanol, which produce the macro-initiator in the form of a

white solid (5.0g, 86.0%). ^{1}H NMR (400 MHz, $CDCl_3$, ppm): δ= 1.93 (s, 6H) 4.33-4.31 (m, 2H), 3.37 (s, 3H). $M_{n,GPC}$= 2300 g/mol.

Cell Viability

Non-cancerous HEK-293 (normal cell) and BT-20 (cancer cell) were purchased from the ATCC (Manassas, USA). The cells were grown in the respective medium [HEK-293: DMEM (Gibco) + 15% FBS (Gibco) + AA (Gibco); BT-20: RPMI (Gibco) + 10% FBS (Gibco) + AA (Gibco)], and then the cells were maintained in a humidified CO_2 incubator at 37 °C with 5% CO_2. The biocompatibility of non-CCL and C-C/S-S/Se-Se CCL micelles on HEK-293 cells as well as the cytotoxic impact of DOX-loaded C-C/S-S/Se-Se CCL micelles on the BT-20 cancer cells were determined using a cell viability test. HEK-293 normal cells were treated with different doses of non-CCL and C-C/S-S/Se-Se CCL micelles (0, 25, 50, 100, 200 μg/mL), whereas BT-20 cells were treated with various doses of C-C/S-S/Se-Se CCL/DOX micelles and free DOX (0, 2.5, 5.0, 10, 20 and 30 μg/mL of DOX concentration) and incubated for 24 h. The cytotoxicity assays were conducted using WST assay according to the manufacturer's protocol.

Confocal Microscopy

The BT-20 cells were cultured on sterile coverslips (density: 5.0×10^4 cells/well) settled in a confocal dish, treated with 30 μg/mL DOX concentration of C-C/S-S/Se-Se CCL/DOX micelles, and free DOX for 12 h and 24 h. The BT-20 cells were rinsed two times with a PBS buffer and fixed for 15 min with a paraformaldehyde (4% in PBS) solution. The BT-20 cells were washed in PBS, stained with a DAPI (1 mg/mL in PBS) solution, and incubated at 37°C for 10 minutes. A Fluorescence Confocal Microscope was used to investigate the nuclear morphology of BT-20 cells (Carl Zeiss, Oberkonchen, Germany).

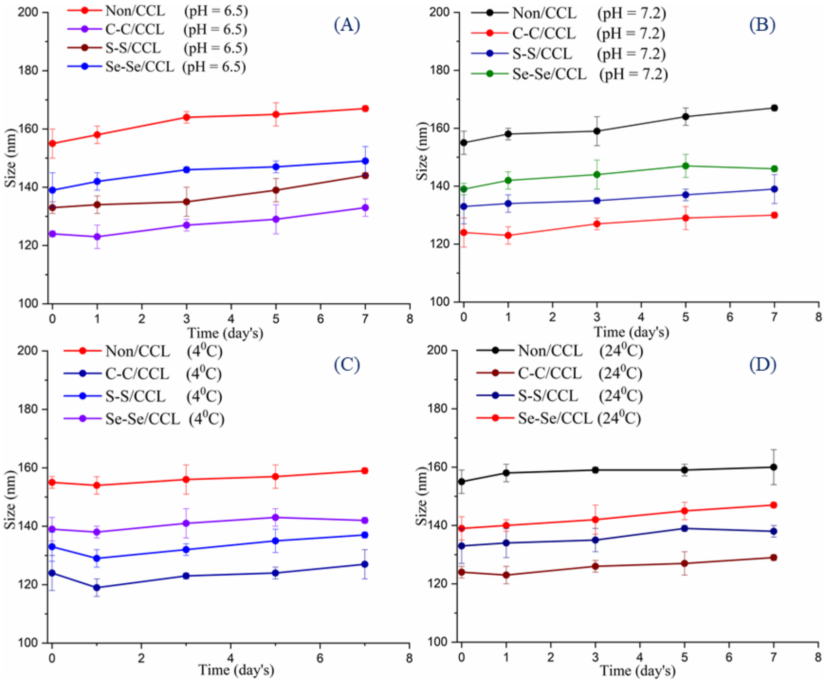

Figure S1. Stability of C-C/S-S/Se-Se CCL and non-CCL micelles at pH = 6.5 (A) and pH = 7.2 (B) and 4^0C (C), 24^0C (D).

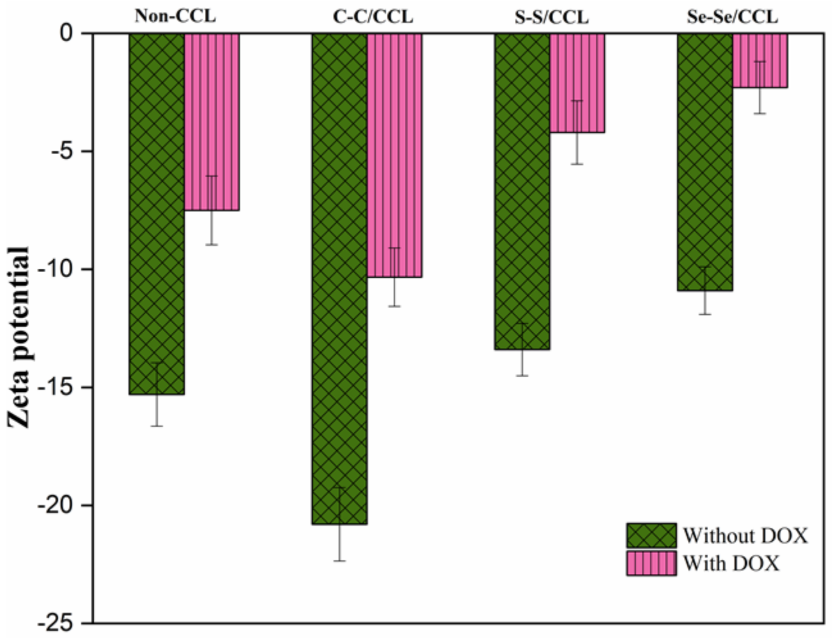

Figure S2. Zeta potential measurement of Non-CCL and C-C/S-S/Se-Se CCL micelles with and without DOX.

Reference

1) Tufail, M.K.; Abdul-Karim, R.; Rahim, S.; Musharraf, S.G.; Malik, M.I. Analysis of individual block length of amphiphilic di-& tri-block copolymers containing poly (ethylene oxide) and poly (methyl methacrylate). *RSC Adv*. **2017**, 7, 41693-41704.
